# Mutations in topoisomerase IIβ result in a B cell immunodeficiency

**DOI:** 10.1038/s41467-019-11570-6

**Published:** 2019-08-13

**Authors:** Lori Broderick, Shawn Yost, Dong Li, Matthew D. McGeough, Laela M. Booshehri, Marisela Guaderrama, Susannah D. Brydges, Karolina Kucharova, Niraj C. Patel, Margaret Harr, Hakon Hakonarson, Elaine Zackai, Ian G. Cowell, Caroline A. Austin, Boris Hügle, Corinna Gebauer, Jianguo Zhang, Xun Xu, Jian Wang, Ben A. Croker, Kelly A. Frazer, Christopher D. Putnam, Hal M. Hoffman

**Affiliations:** 1 Department of Pediatrics, University of California at San Diego, La Jolla, CA 92093 USA; 2 Rady Children’s Hospital of San Diego, San Diego, CA 92123 USA; 3 Division of Genetics and Epidemiology, Institute of Cancer Research, London, SM2 5NG UK; 4 Center for Applied Genomics, Children’s Hospital of Philadelphia, Philadelphia, PA 19104 USA; 5 Department of Medicine, University of California at San Diego, La Jolla, CA 92093 USA; 6 Regeneron Pharmaceuticals, Inc., Tarrytown, NY 10591 USA; 7 Laboratory of Molecular Immunology, Sanford Burnham Prebys Medical Discovery Institute, La Jolla, CA 92093 USA; 8 Department of Pediatrics, Levine Children’s Hospital, Atrium Health, Charlotte, NC 28203 USA; 9 Department of Genetics, Children’s Hospital of Philadelphia, Philadelphia, PA 19104 USA; 10 Department of Pediatrics, University of Pennsylvania School of Medicine, Philadelphia, PA 19104 USA; 11 Institute for Cell and Molecular Biosciences, Newcastle University, Newcastle upon Tyne, NE2 4HH UK; 12 German Center for Pediatric and Adolescent Rheumatology, Garmisch-Partenkirchen, 82467 Germany; 13 Division of Neonatology, University Hospital for Children and Adolescents, University of Leipzig, Leipzig, 04109 Germany; 14 BGI-Shenzhen, Beishan Industrial Zone, Shenzhen, 518083 China; 15 China National GeneBank, BGI-Shenzhen, Jinsha Road, Shenzhen, 518120 China; 16 Institute for Genomic Medicine, University of California San Diego, La Jolla, CA 92093 USA; 17 San Diego Branch, Ludwig Institute of Cancer Research, La Jolla, CA 92093 USA

**Keywords:** Immunological disorders, B cells, Medical genetics, Immunological deficiency syndromes

## Abstract

B cell development is a highly regulated process involving multiple differentiation steps, yet many details regarding this pathway remain unknown. Sequencing of patients with B cell-restricted immunodeficiency reveals autosomal dominant mutations in *TOP2B*. *TOP2B* encodes a type II topoisomerase, an essential gene required to alleviate topological stress during DNA replication and gene transcription, with no previously known role in B cell development. We use *Saccharomyces cerevisiae*, and knockin and knockout murine models, to demonstrate that patient mutations in *TOP2B* have a dominant negative effect on enzyme function, resulting in defective proliferation, survival of B-2 cells, causing a block in B cell development, and impair humoral function in response to immunization.

## Abstract (web summary)

Topoisomerases are required to release topological stress on DNA during replication and transcription. Here, Broderick et al. report genetic variants in *TOP2B* that cause a syndromic B cell immunodeficiency associated with reduced TOP2B function, defects in B cell development and B cell activation.

## Introduction

DNA replication and gene transcription require that topological stress be relaxed; a critical cellular function performed by topoisomerases. Humans possess two genes that encode type II topoisomerases, *TOP2A* and *TOP2B*, which make transient double-stranded DNA breaks that alleviate negative supercoils^1^. Failure to relax topological stress can reduce the production of multiple gene products, even in the absence of a genetic mutation. These defects preferentially cause loss of function of large genes, which are most vulnerable to topologic stress^2,3^. To our knowledge, no monogenic disease has been linked to the inability to relax topological stress.

The study of rare immunodeficiency patients has been extremely instructive to our understanding of B-cell development and human disease, revealing mechanisms of B-cell development, central tolerance, and protection from infection^4–7^. These immunodeficiencies are primarily characterized by their effects on the B-cell compartment and typically X-linked recessive (X-linked agammaglobulinemia) or autosomal recessive^7^. In contrast, syndromic immunodeficiencies are a distinct subset of childhood primary immunodeficiencies in which the physical malformations are often more pronounced than the underlying immunologic defects, leading to delays in diagnosis. Chromosomal abnormalities and metabolic defects have been hypothesized to underlie many multisystem syndromic diseases^8^. However, the identification of single gene in such disorders suggests they likely affect a shared cellular process that impacts the development of multiple organ systems.

We previously described two unrelated families with autosomal dominant inherited syndromic B-cell immunodeficiency of unknown etiology^9,10^. While agammaglobulinemia and hypogammaglobulinemia are common to several classic immunodeficiencies, the patients were also affected by facial dysmorphism, and limb anomalies, identifying this as a unique syndrome, subsequently named Hoffman syndrome, but the genetic etiology remained unknown until now.

Here, we show that mutations in *TOP2B* underlie the syndromic B-cell immunodeficiency and investigate how these dominant genetic defects lead to reduced TOP2B function, defects in B-cell development, and B-cell activation in response to antigen stimulation using models in *Saccharomyces cerevisiae*, and knockout and knockin murine lines. These results are the initial description of a role for *TOP2B* in human immunodeficiency syndromes associated with impaired B-cell development and function.

## Results

### Identification of TOP2B mutations

**Fig. 1:**
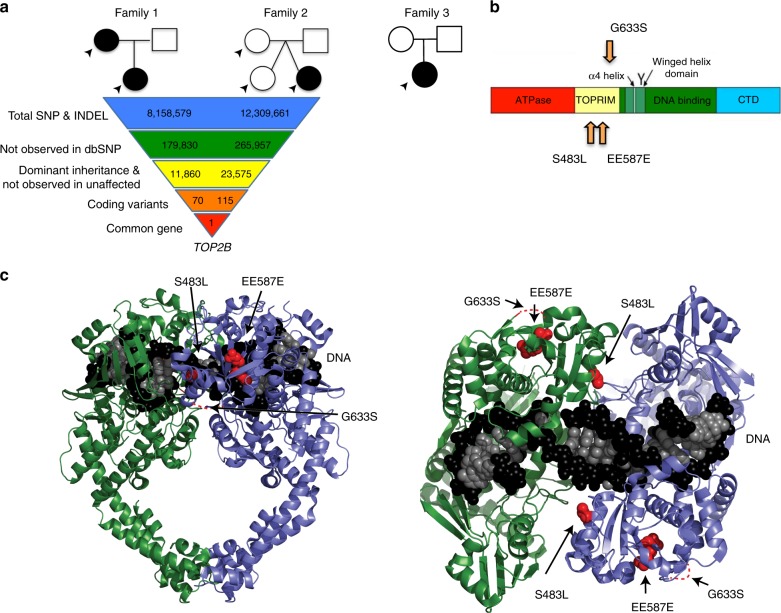
Mutations in *TOB2B* cause peripheral B-cell immunodeficiency and dysmorphic features. **a** Pedigrees in three unrelated families with variants in *TOP2B*. Filled symbols, subjects with mutation; open symbols, unaffected subjects. Arrows indicate individuals subjected to whole genome sequencing. Three variants were identified: a missense mutation c.1448C > T, p.S483L, a de novo inframe deletion c.1761_1763AGAdel p.EE587E, a missense mutation c.1897G > A, p. G633S. **b** Modeling of the identified variants in protein domains. Linear modeling shows the approximate location of patient mutations in the TOPRIM domain. **c** 3D modeling with green and blue helices representing two domains of the homodimer, and black and white spheres representing the DNA helix in the binding site. Mutations are displayed in red in the structure of the TOPRIM domain

We previously reported two unrelated families with autosomal dominant inherited syndromic B-cell immunodeficiency of unknown etiology (Table 1, Supplementary Table 1)^9,10^. The probands from each family presented with recurrent infections by polysaccharide-encapsulated bacteria, severe hypogammaglobulinemia, and absent CD19+ B cells, but had normal T-cell responses to mitogens. We performed whole genome sequencing of affected and nonaffected persons in these families (Fig. 1a). We filtered variants within each family assuming that the variants would not be in dbSNP^11^, would have a dominant inheritance pattern, and would affect coding sequences (Supplementary Tables 2–4). One gene, *TOP2B*, which encodes topoisomerase IIβ^12,13^ was affected by a heterozygous variant in each family: a de novo in-frame deletion c.1761_1763delAGA, p.EE587E, and a missense mutation c.1448C > T, p.S483L. A third patient initially being reported as having an atypical presentation of Ablepharon-Macrostomia syndrome^14^, was later found to have humoral immunodeficiency. Independent reanalysis of clinical whole-exome sequencing revealed a heterozygous *TOP2B* variant: c.1897G > A, p.G633S. All of the mutations affected the TOPRIM domain of TOP2B (Fig. 1b)^15^. Notably, *TOP2B* has a probability of loss of function intolerance (pLI) of 1 (Supplementary Table 5), similar to most known severe haploinsufficiency human disease genes^16^, suggesting that the identified heterozygous mutations in our patients could cause disease either through haploinsufficiency or a partial genetic dominance.

**Table 1:** Immunologic laboratory values at time of diagnosis of patients with *TOP2B* mutations^a^

	Family 1		Family 2	Family 3
Age at presentation	7 years	35 years	6 years	5 years
*General immune evaluation*				
Leukocytes (cells/μL)	5200	7600	7000	5900
	[4500–13500]	[4000–1000]	[5500–15,000]	[5500–15,000]
Absolute Lymphocyte count (cells/μL)	1900	2000	2240	3547
	[1500–6500]	[1200–3500]	[1500–7000]	[1500–7000]
CD3+ T cells (cells/μL)	1853	1764	2720	3422
	[1200–2700]	[1100–2500]	[1900–3000]	[1400–3700]
CD4+ T cells (cells/μL)	1122	1008	1728	1832
	[800–1500]	[700–1400]	[570–1200]	[700–2200]
CD8+ T cells (cells/μL)	774	726	800	1417
	[400–1200]	[400–1100]	[475–960]	[490–1300]
CD19+ B cells (cells/μL)	0	48	0	0
	[150–600]	[150–500]	[200–600]	[200–600]
CD16+ NK cells (cells/μL)	58	172	448	17
	[200–400]	[150–400]	[200–600]	[200–600]
CH50	n.d.	n.d.	90	>65
	[n.a.]	[n.a.]	[>80]	[>60]
*B cell-specific immune evaluation*				
Total IgG (mg/dL)	232	631	73	242
	[610–1480]	[720–1460]	[400–1075]	[463–1236]
IgA (mg/dL)	<26	158	33	7
	[40–290]	[24–381]	[19–119]	[25–154]
IgM (mg/dL)	150	128	12	<6
	[380–1860]	[54–227]	[25–114]	[43–196]
IgE (mg/dL)	n.d.	n.d.	5	n.d.
	[n.a.]	[n.a.]	[0–450]	[n.a.]
tetanus titer^b^ (IU/mL)	deficient	n.d.	<0.04–> 0.11	0.1
	[>0.15]	[n.a.]	[>0.15]	[>0.15]
Diphtheria (IU/mL) titers	deficient	n.d.	undetectable	<0.1
	[>0.15]	[n.a.]	[>0.15]	[>0.15]
H. influenzae B titers (ng/mL)	deficient	n.d.	undetectable	<0.15
	[>0.15]	[n.a.]	[>100]	[>0.15]
Mitogen stimulation	normal	normal	normal	normal

^a^Normal values for age, per reference lab, shown in square brackets. n.d. not done, n.a. not available

^b^Titer values are expressed as expected postimmunization^9,10^

### Patient mutations inactivate topoisomerase II function

The patient mutations affected highly conserved amino acids (Supplementary Fig. 1). We tested the ability of patient *TOP2B* mutations to complement the temperature sensitivity of the *top2–4* allele of *S. cerevisiae TOP2*, an essential gene that encodes the only *S. cerevisiae* topoisomerase II homolog^17^. We introduced the *S. cerevisiae* equivalents of the patient mutations into a previously established plasmid encoding a chimeric *S. cerevisiae–*human *TOP2B* fusion, which can complement the *top2–4* growth defect at 37 °C^18^. Unlike the wild-type construct, chimeric constructs containing the patient mutations S483L or EE587E were unable to complement *top2–4* at the nonpermissive temperature, similar to an empty vector control (Supplementary Fig. 2).

**Fig. 2:**
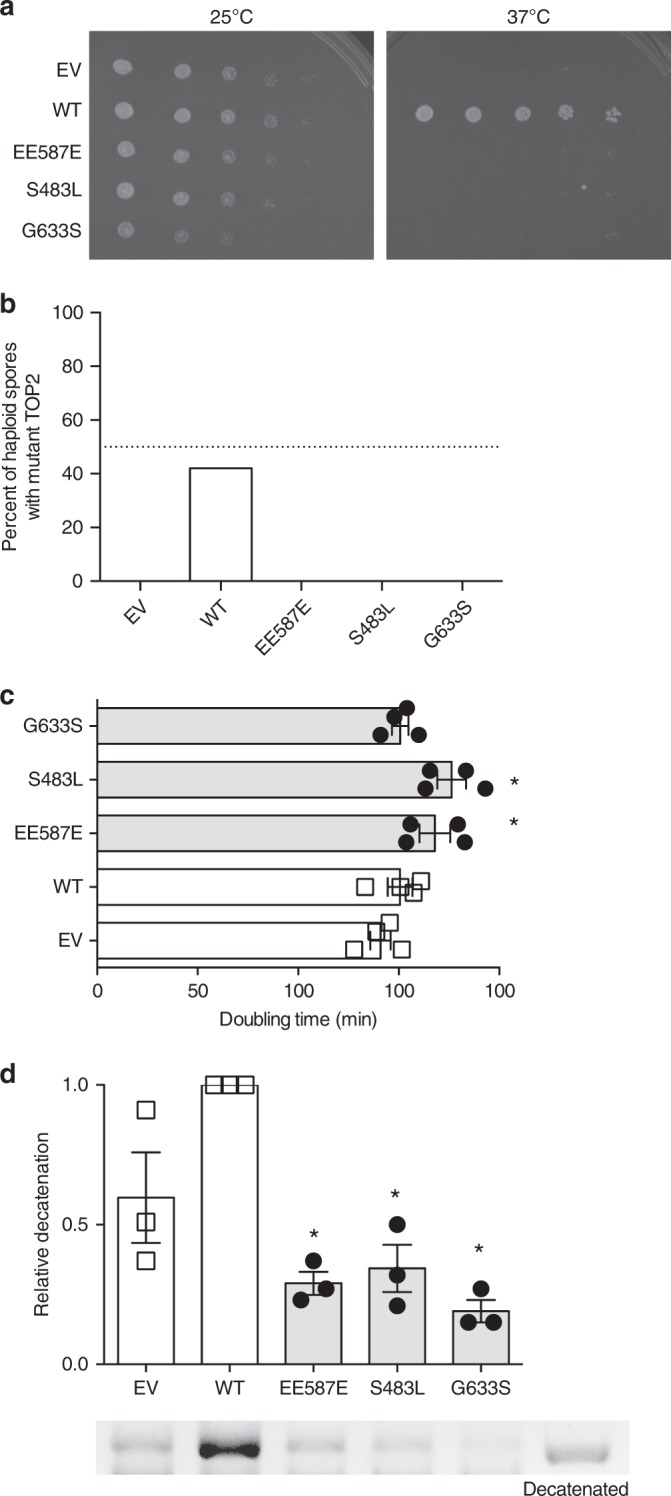
Patient mutations have a dominant negative phenotype in *S. cerevisiae*. **a** The temperature-sensitive *top2–4 S. cerevisiae* strain JN394t2–4 was transformed with a wild-type *TOP2* vector (WT), mutant *TOP2* vectors, or an empty vector (EV). Serial dilutions of transformants were spotted and incubated at 25 °C (left) or 37 °C (right). The disease-associated mutations and the empty vector were unable to complement the *top2–4* mutation. Selection plates shown are representative of three experiments of independent *S. cerevisiae* clones. **b** Mutant *top2* alleles were not viable. The diploid strain was sporulated, and random spores grown at 30 °C and scored for the presence of *top2Δ*. *top2Δ* spore clones were not recovered with diploids containing the empty vector and ScTOP2-EE587E, -S483L, and -G633S vectors (dotted line indicates 50%, Source data in Supplementary Table 6). **c** Haploid strains heterozygous for syndrome-associated *TOP2* mutations (ScTOP2-EE587E and ScTOP2-S483L) have increased doubling time, whereas strains carrying ScTOP2-G633S were not as severely affected. (*n* = 5 independent experiments, shown as mean ± SEM, *p < 0.05, one-way ANOVA). **d** Nuclear fractions containing Top2 isolated from haploid heterozygous strains with ScTOP2-EE587E, -S483L, and -G633S did not decatenate kinetoplast DNA as effectively as controls containing two copies of wild-type ScTOP2 or one copy of wild-type ScTOP2 and an empty vector (representative gel, Source data are provided as a Source Data file; *n* = 3 independent experiments, shown as mean ± SEM, *p < 0.05, Kruskall–Wallis, compared to WT)

To ensure that patient mutations were not interacting with some aspect of the Top2–TOP2B protein chimera, we constructed a low-copy number plasmid containing the *S. cerevisiae TOP2* gene (and its native promoter) that contained an artificial intron to avoid toxicity in *Escherichia coli* strains (ScTOP2 vector, Supplementary Fig. 3). Similar to the mutant chimeric constructs but unlike the wild-type ScTOP2 plasmid, the mutant ScTOP2-S483L, -EE587E, and -G633S vectors were unable to complement the *top2–4* at the nonpermissive temperature, consistent with a loss of function (Fig. 2a).

To demonstrate that the disease-associated mutations were inactive and not just temperature sensitive, we sporulated a heterozygous *top2Δ/TOP2* diploid *S. cerevisiae* strain that contained either wild-type or mutant versions of the ScTOP2 vector. We found that the wild-type ScTOP2 vector allowed robust recovery of *top2Δ*-containing haploids (Fig. 2b, Supplementary Table 5). No haploid spore clones lacking *TOP2* were recovered with an empty vector control or with the ScTOP2-EE587E, -S483L, or -G633S vectors (Fig. 2b). Taken together, these results indicate that each of our patient mutations disrupt topoisomerase II function.

### Mutations have a partial dominant phenotype in *S. cerevisiae*

The effects of the heterozygous patient *TOP2B* mutations could be due to haploinsufficiency or to the formation of partially inactive wild-type/mutant TOP2B heterodimers. To test these possibilities, we isolated a *top2Δ* haploid containing the wild-type ScTOP2 vector and introduced a second vector; both vectors were present at the same copy number (Supplementary Fig. 4a). Growth analysis demonstrated that the strains with ScTOP2-EE587E or ScTOP2-S483L grew more slowly than the wild-type controls (Fig. 2c), but were not associated with the activation of the DNA damage checkpoint (Supplementary Fig. 4b). Nuclear fractions derived from these strains were tested for Top2-dependent decatenation activity. Remarkably, fractions from the strain with only one copy of wild-type *TOP2* decatenated kinetoplast DNA more effectively than fractions from strains with one wild-type and one mutant copy of *TOP2* (Fig. 2d, Supplementary Fig. 4c). Thus, the disease-associated versions of *TOP2* have a partial dominant negative phenotype in *S. cerevisiae* based on growth and/or decatenation assays.

### Loss of *TOP2B* negatively affects the B-cell compartment

**Fig. 3:**
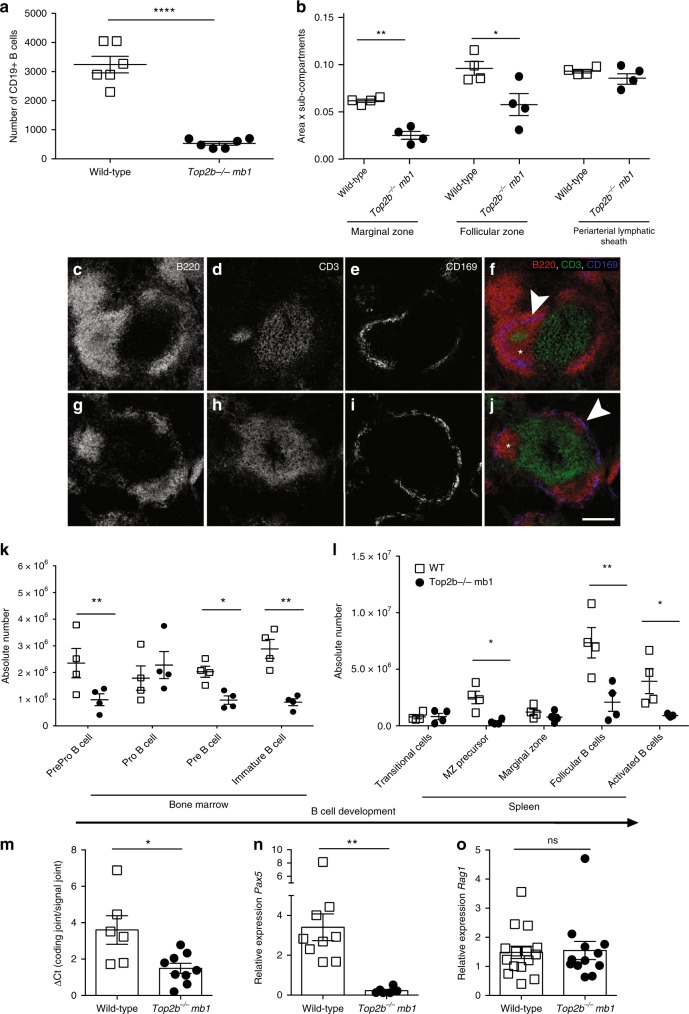
B-cell development is negatively affected in the absence of *TOP2B*. **a** Absence of *TOP2B* in B cells results in reduced CD19+ B cells in peripheral blood compared to littermates (*n* = 6 WT and 7 *Top2b*^*−/−*^ *mb1 cre* mice per group, mean ± SEM, ****p < 0.0001). **b** Splenic white pulp subcompartments are reduced in size in the absence of Top2b. Area of three sub-compartments were evaluated in at least ten follicles across three sections (500 μM apart) with marker indicating average per mouse. (Mean SEM, *p < 0.05). Representative follicles from wildtype (**c**–**f**) and *Top2b*^*−/−*^ *mb1*-*cre* spleens (**g**–**j**) show altered splenic architecture. More B220-expressing (red) cells are seen in the wildtype (**c**, **f**) than *Top2b*^*−/−*^ *mb1*-*cre* mice (**g**, **j**). The metallophilic macrophages (blue) indicating the border between marginal zone and follicular region. The periarteriolar lymphoid sheath (PALS) subcompartment contains T cells (green). Scale bar indicates 200 µm. Bone marrow (**k**) and spleen (**l**) cells were isolated from wildtype and *Top2b*^*−/−*^ *mb1-cre* mice. Flow cytometry demonstrates that the bone marrow of *Top2b*^*−/−*^ *mb1-cre* mice have fewer PrePro B cells (Fr. A, B220+, CD43−, BP1−, CD24−), Pre B cells (Fr. D, B220+, CD43−, IgD−, IgM−), and immature B cells (Fr. E, CD19+, B220+, CD43−, IgM+, IgD−) compared to controls (**k**). In the spleen (**l**), there are overall fewer B cells with reductions in marginal zone precursors (CD19+, B220+, IgMhi, IgDhi, CD21/35+), follicular (CD19+, B220+, IgMlo, IgDhi, CD38+), and activated (CD19+, B220+, IgDhi, IgMhi, Flt3+) B cells compared to controls. (*n* = 4 mice per group, mean ± SEM, absolute numbers calculated from whole tissue numbers). *p < 0.05; **p < 0.01. **m** qPCR analysis of kappa-deleting recombination excision circles demonstrate that B cells isolated from *Top2b*^*−/−*^ *mb1-cre* mice have reduced replication in the spleen (*n* = 6 WT or 9 *Top2b*^*−/−*^ *mb1 cre* mice, shown as mean ± SEM). *p < 0.05. **n** *Pax5* is significantly decreased in B cells isolated from *Top2b*^*−/−*^ *mb1-cre* mice (*n* = 9 WT or 6 *Top2b*^*−/−*^ *mb1- cre* mice), whereas *Rag1* expression (**o**) is unchanged (*n* = 5 WT and 4 *Top2b*^*−/−*^ *mb1- cre *mice per group shown as mean ± SEM, with technical triplicates), **p < 0.005). All by two-tailed Student’s *t* test

Affected patients have a normal T-cell compartment, but lack B cells in the peripheral blood^1,2^. Notably, patients also have dramatically reduced but detectable levels of circulating immunoglobulins (Table 1), suggesting that B cells in the patients are substantially reduced, but may not be entirely lost. To test the role of *TOP2B* in B-cell development, we bred *Top2b*^*flox2*^ mice to two independent B cell-specific cre mice: *cd19-cre*^19^ and *mb1-cre*^20^, as *Top2b*^*−/−*^ mice do not survive the perinatal period^21^. Flow cytometry of peripheral blood demonstrated that *Top2b*^*−/−*^*mb1-cre* and *Top2b*^*−/−*^*cd19-cre* mice had fewer CD19+ B cells than wild-type controls (Fig. 3a, Supplementary Fig. 5a). The *Top2b*^*−/−*^*cd19-cre* mice were less severely impaired, suggesting that Top2b plays a role earlier in the B-cell lineage than is affected by the *cd19-cre* transgene^20^. Therefore, we focused on our *Top2b*^*−/−*^*mb1-cre* model.

Immunocytochemical evaluation of *Top2b*^*−/−*^*mb1-cre* mice showed altered splenic follicle structure, with reduced marginal zone and follicular B-cell zones (Fig. 3b–j), suggesting that Top2b promotes B-cell development. To further characterize these differences, we examined spleen and bone marrow from *Top2b*^*−/−*^*mb1-cre* and control mice for conventional B cells by flow cytometry. Immunophenotyping demonstrated that the bone marrow of *Top2b*^*−/−*^*mb1-cre* mice have fewer PrePro B cells, Pre B, and immature B cells compared to control mice (Fig. 3K). In the spleen, there were fewer B cells at every developmental stage compared to control mice (Fig. 3l). Analysis of kappa-deleting recombination excision circles (KRECs)^22^ demonstrated that B cells derived from *Top2b*^*−/−*^*mb1-cre* mice had fewer KRECs compared to controls in the spleen and bone marrow (Fig. 3m and Supplementary Fig. 5b), suggesting a reduced replication history. Furthermore, expression of *Pax5*, a B-cell specific transcription factor expressed at most stages of B-cell development^23–25^, was significantly reduced in B cells derived from *Top2b*^*−/−*^*mb1-cre* mice while *Rag1* expression was unchanged (Fig. 3n, o). Together, these data suggest B-cell specific defects caused by the absence of Top2b may occur due to transcriptional defects affecting multiple stages of B-cell development.

### B cells fail to activate normally in the absence of Top2b

**Fig. 4:**
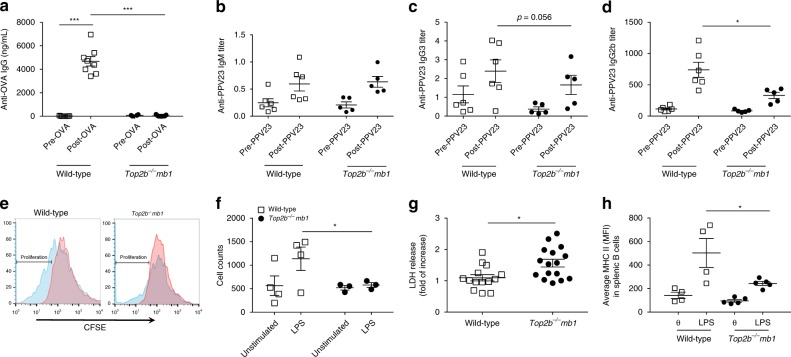
B cells are functionally impaired in the absence of Top2b, in vivo and in vitro. **a** Mice fail to produce specific IgG antibody in response to vaccination with ovalbumin (OVA) in the absence of Top2b in B cells (*n* = 9 mice per group, shown as mean ± SEM, ***p < 0.005 by the Kruskal–Wallis test). **b**–**d** Adult *Top2b*^*−/−*^ *mb1 cre* and littermate mice were immunized with Pneumovax-23 (PPV23). At 6 days post-immunization (**b**), B cells produce PPV23-specific IgM in the absence of TOP2B, at similar levels to wild-type control mice. Four weeks after immunization with Pneumovax-23, anti-PPV23 IgG3 (**c**) and IgG2b (**d**) production is impaired in *Top2b*^*−/−*^ *mb1-cre* mice compared to wild-type control animals (*n* = 6 WT or 5 *Top2b*^*−/−*^ *mb1 cre*, shown as mean ± SEM, *p < 0.05 by the Kruskal–Wallis test). **e** In vitro, *Top2b*^*−/−*^ *mb1 cre* mouse-derived B cells fail to proliferate, as assessed by CFSE dilution, in the presence of stimulation with LPS (blue histogram). Untreated, CFSE stained cells are shown in red. FACS plots are representative of 4 mice per group. Total number of CFSE positive cells is quantified in (**f**). **g** *Top2b*^*−/−*^ *mb1*-*cre* mouse-derived B cells have increased cell death as measured by LDH release (*n* = 7 WT or 8 *Top2b*^*−/−*^ *mb1 cre* mice per group, in duplicate, shown as mean ± SEM). *p < 0.05, two-tailed Student’s *t* test. **h** The B cells that do survive have reduced upregulation of the activation marker MHC II, as shown by mean fluorescence intensity (MFI). *p < 0.05, two-tailed Student’s *t* test (*n* = 4 mice per group)

To test the role of *Top2b* in humoral immunodeficiency and antigen-specific responses, *Top2b*^*−/−*^*mb1-cre* and littermate control mice were immunized with ovalbumin (OVA). Fourteen days after immunization, serum samples from control mice demonstrated increased OVA-specific IgG, which was not observed in *Top2b*^*−/−*^*mb1-cre* mice (Fig. 4a). To determine if this defect was OVA-specific, mice were immunized with polysaccharide antigen Pneumovax-23 (PPV23)^26,27^. There was no difference in PPV23 binding IgM at zero or 6 days post-immunization between *Top2b*^*−/−*^*mb1-cre* and control mice, and B-1 cells in the bone marrow and spleen were unaffected (Fig. 4c, Supplementary Fig. 5c, d). Similar to OVA immunization, anti-PPV23 IgG3 trended downwards, and anti-PPV23 IgG2b was significantly reduced in *Top2b*^*−/−*^*mb1-cre* mice compared to controls at 6 weeks post-immunization (Fig. 4c, d). The immunization responses in the *Top2b*^*−/−*^*mb1-cre* mice are consistent with the failure to mount a vaccine response observed in patients.

Since the antigen-specific responses could reflect depletion of B cells in *Top2b*^*−/−*^*mb1-cre* mice in addition to any B-cell activation defects, splenic B cells were isolated and stimulated in vitro with lipopolysaccharide (LPS). After 72 h of stimulation, *Top2b*^*−/−*^ *mb1-cre* derived B cells failed to proliferate and had increased cell death compared to their wild-type counterparts (Fig. 4e–g). In addition, surviving *Top2b*^*−/−*^ *mb1-cre* B cells had less robust upregulation of the activation marker MHC II (Fig. 4h). Together these results suggest that the defects in antigen-specific responses in the absence of Top2b are due to both reduced numbers of B cells as well as defects in the secretion of antibody or in the generation of mature, activated B cells.

### Mutant *Top2b* negatively affects B-cell development in mice

**Fig. 5:**
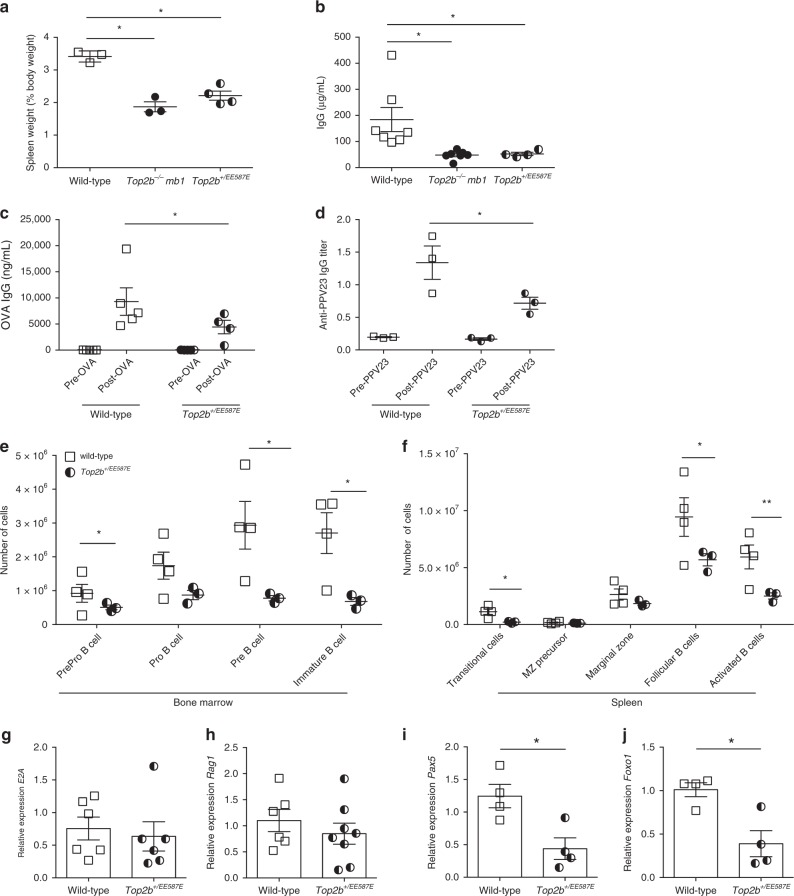
B cell development is negatively affected when*TOP2B* is mutated. **a** Spleen to body weight ratio for wild-type (*n* = 3), *Top2b*^*−/−*^*-mb1 cre* (*n* = 3), and *Top2b*^*+/EE587E*^ mice (*n* = 4) reveal smaller ratios in knockin and knockout mice compared to controls (shown as mean ± SEM). *p < 0.05 by Kruskal–Wallis test. **b** Total serum IgG is reduced in *Top2b*^*−/−*^ *mb1 cre* mice (*n* = 6) and *Top2b*^*+/EE587E*^ (*n* = 4) mice compared to controls (*n* = 7, shown as mean ± SEM). *p < 0.05 by Kruskal–Wallis test. **c**, **d** Heterozygous mutant mice fail to produce specific IgG antibody in response to vaccination with ovalbumin (OVA, *n* = 4, (**c**) or pneumovax-23 (**d**) (*n* = 3 mice per group, shown as mean ± SEM, *p < 0.05 by the Kruskal–Wallis test). Bone marrow (**e**) and spleen (**f**) cells were isolated from *Top2b*^***+/****EE587E*^ mice and littermate controls. Phenotypic analysis by flow cytometry demonstrates that the bone marrow of *Top2b*^*+/EE587E*^ mice (*n* = 3) have fewer PrePro B cells (Fr. A, B220+, CD43−, BP1−, CD24−), Pre B cells (Fr. D, B220+, CD43−, IgD−, IgM−), and immature B cells (Fr. E, CD19+, B220+, CD43−, IgM+, IgD−) compared to control mice (**e**). In the spleen (**f**), there are overall fewer B cells with significant reductions in transitional (CD19+, B220+, IgDlo, IgM+, CD21/35int, CD23 variable), follicular (CD19+, B220+, IgMlo, IgDhi, CD38+) and activated (CD19+, B220+, IgDhi, IgMhi, Flt3+) B cells compared to control mice (*n* = 4, shown as mean ± SEM, absolute numbers calculated from whole tissue numbers). *p < 0.05; **p < 0.01, two-tailed Student’s *t* test. Expression of mutant *Top2b* results does not affect *E2A* (**g**, *n* = 6)) or *Rag1* (**h**, *n* = 8) expression in common lymphoid progenitor cells (compared to WT *n* = 6), whereas B cell-specific transcription factors *Pax5* (**i**) and *Foxo1* (**j**) show reduced expression of compared to wild-type mice. (*n* = 4 mice per group, shown as mean ± SEM, *p < 0.05, two-tailed Student’s *t* test)

To evaluate the effects of mutant Top2b expression on the B-cell compartment in mice, we generated a constitutive knockin mouse carrying the murine equivalent of the *TOP2B* mutation EE587E. *Top2b*^*EE587E/EE587E*^ homozygous mice were not viable, similar to *Top2b*^*−/−*^ mice, but *Top2b*^*+/EE587E*^ heterozygotes could be recovered, which mirror the genotype of the Hoffman syndrome patients (Supplementary Table 7). Several observations indicated the presence of B cell defects in the *Top2b*^*+/EE587E*^ mice relative to wild-type controls: (1) the spleen-to-body ratio was reduced (Fig. 5a); (2) total serum IgG was reduced (Fig. 5b); (3) immunization with ovalbumin or Pneumovax-23 generated significantly lower titers of antigen-specific antibody (Fig. 5c, d); and (4) flow cytometric immunophenotyping of the bone marrow and spleen demonstrated reduced numbers of B cells at most stages of B-cell development (Fig. 5e, f, Supplementary Fig. 6a). Thus, even heterozygous expression of the patient-derived *Top2b* mutation had deleterious effects on the B-cell compartment that were comparable to the effect of the *Top2b*^*−/−*^*mb1-cre* mice.

Patients have normal numbers of CD4 and CD8 T cells, but no detectable peripheral B cells in blood (Table 1), despite cells originating from a common lymphocyte precursor. Since defects in *TOP2B* give rise to reduced transcription of long genes^2,3^, we investigated the expression of transcription factors involved in establishing and/or maintaining B- and T-cell fate. *Rag1* and *E2A*, genes required for both B- and T-cell development, were similarly expressed in bone marrow derived lineage-negative CD117-negative progenitor cells between mutant and wild-type mice (Fig. 5g, h). However, pre-B cells from *Top2b*^*+/EE587E*^ mice demonstrated reduced expression of the B cell-specific transcription factors *Pax5* and *Foxo1* (Fig. 5I, J). Unlike the B cell-specific transcription factors, expression of the T cell-specific transcription factors *Notch1* and *Tcf1* in the spleen was similar to littermate controls (Supplementary Fig. 6b, c).

### Mutant *Top2b* has a dominant negative effect on B cells

**Fig. 6:**
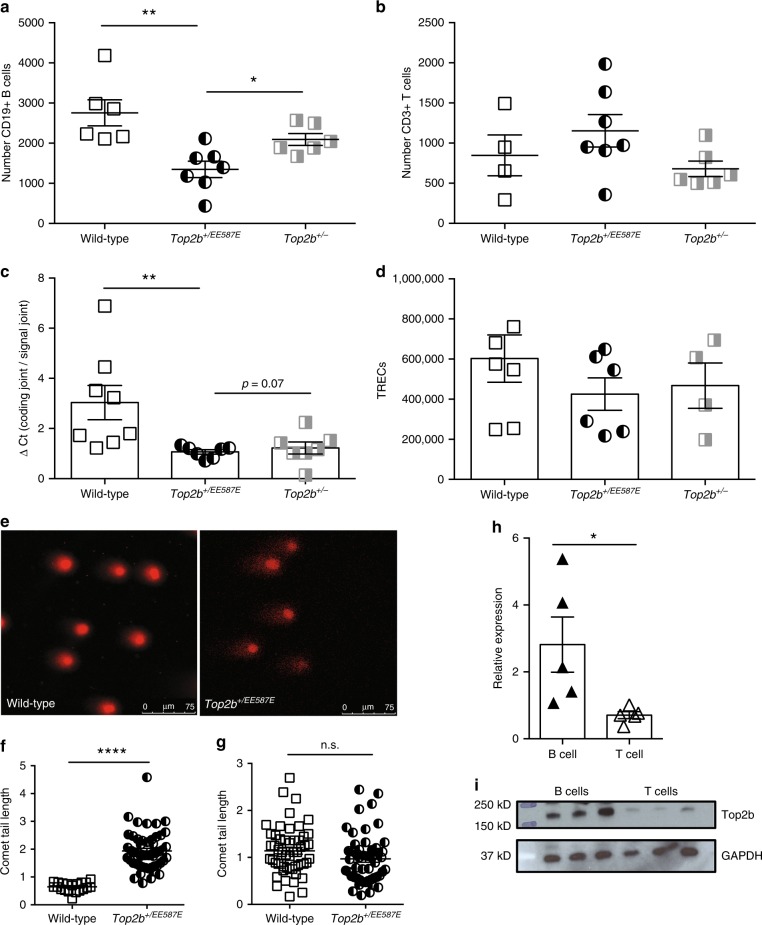
Heterozygous mutation in *Top2b* negatively affects B-cell development beyond haploinsufficiency. Expression of mutant *Top2b* (*n* = 7) results in reduced CD19+ B cells (**a**) in peripheral blood compared to wild-type (*n* = 6) or hemizygous mice (*n* = 6), while T cells (**b**) are unaffected.(shown as mean ± SEM, *p < 0.05, **p < 0.005, two-tailed Student’s *t* test). **c** qPCR analysis of kappa-deleting recombination excision circles demonstrates that B cells isolated from *Top2b*^*+/EE587E*^ (*n* = 7) or *Top2b*^*+/−*^ mice (*n* = 7) have a less robust replication history compared to wild-type mice (*n* = 8 mice), shown as mean ± SEM, while T-cell proliferation as measured by T-cell receptor excision circle (TREC) formation is unaffected (**d**), (*n* = 6 WT, 6 mutant and 4 hemizygous mice, performed with technical duplicates shown as mean ± SEM). *p < 0.05, **p < 0.005, one-way ANOVA. **e**–**g** Comet assay shows increased DNA strand breaks in B cells (**e**, **f**) isolated from *Top2b*^*+/EE587E*^ mice compared to wildtype, expressed as comet tail length. T cells (**g**) are not affected (*n* = 3 mice per group, 10 images per mouse (X40); *p < 0.05; ****p < 0.0001, by Kruskal–Wallis test (**f**), or nonsignificant (n.s.) by Student’s *t* test (**g**)). **h**, **i** Expression of Top2b is elevated in wild-type B cells compared to wild-type T cells by RT-qPCR (**h**, *n* = 5 mice, performed in technical triplicates, shown as mean ± SEM, *p < 0.05, two-tailed Student’s *t* test), and immunoblot (**i**, protein isolated from B and T cells from three wild-type mice, representative of four experiments; source data are provided as a Source Data file)

To determine if disease phenotype is due to haploinsufficiency or due to dominant negative behavior of the mutation as observed in our *S. cerevisiae* models, we compared *Top2b*^*+/EE587E*^ mice to *Top2b*^*+/*^^−^ mice. Similar to patient proband A, *Top2b*^*+/EE587E*^ mice had decreased peripheral blood B cells, which were reduced even compared to *Top2b*^*+/*^^−^ mice, without an effect on peripheral blood T cells (Fig. 6a, b). In addition, KRECs were reduced in the *Top2b*^*+/EE587E*^ mice, while T-cell receptor excision circles (TRECs) were unaffected (Fig. 6c, d), suggesting reduced proliferation of B cells with equivalent T-cell proliferation.

Since Top2b defects could directly or indirectly give rise to unrepaired DNA breaks, we analyzed cells using a single-cell comet assay. Splenic B cells isolated from *Top2b*^*+/EE587E*^ mice had increased DNA breaks compared to B cells from *Top2b*^*−/−*^ *mb1 cre* and wild-type mice as measured by the increase in comet tail length (Fig. 6e, f), while the comet tail length was not altered in T cells derived from *Top2b* mutants (Fig. 6g). To assess this B cell-intrinsic effect, expression analysis of WT mouse Top2b at the transcript and protein level was performed. B cells have greater expression of Top2b mRNA and protein compared to T cells (Fig. 6h, i), which is consistent with previous reports in both humans and mice^28–31^. Together these results suggest that Top2b is more active in B cells than in T cells.

### Heterozygous mutant B cells fail to secrete antibodies

**Fig. 7:**
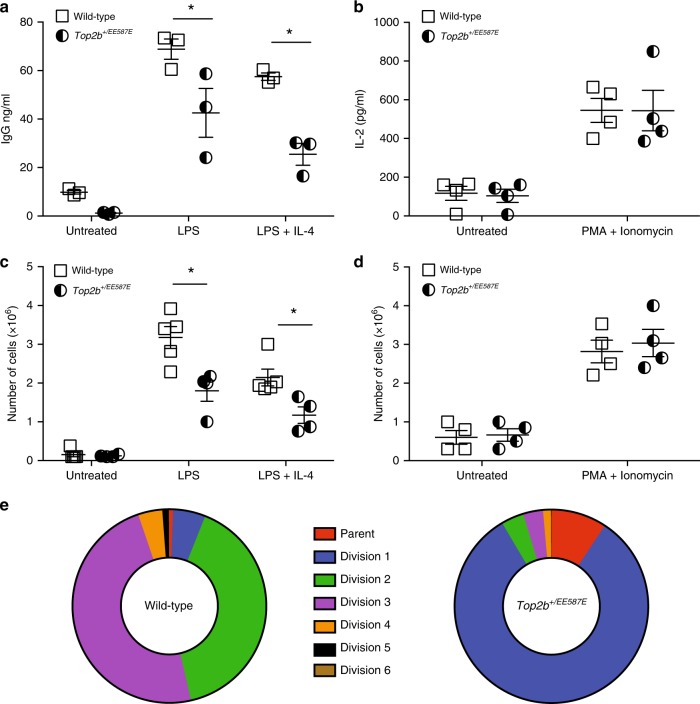
Heterozygous mutant mice fail to respond to in vitro stimulation, while T activation is not affected. Splenic B cells (**a**, **c**) and T cells (**b**, **d**) were isolated from *Top2b*^*+/EE587E*^ and wild-type littermates and stimulated in vitro for measurement of cell counts and protein secretion. Stimulated B cells showed reduced secretion of immunoglobulin in the presence of mutated Top2b (**a**, *n* = 3 mice/group), but T cells secreted IL-2 in equivalent amounts (**b**, *n* = 4 mice per group). **c** Mutant B cells demonstrated reduced proliferation compared to wild-type littermates (*n* = 5 WT and 4 mutant mice per group), whereas T cells (**d**, *n* = 4 mice per group) proliferated similarly between the two groups. (All performed in triplicate. *p < 0.05 by Student’s *t* test). **e** Failure to proliferate is associated with an early arrest. Splenic B cells were isolated from *Top2b*^*+/EE587E*^ and wild-type littermates, stained with CFSE, and stimulated in vitro LPS (25 μg/mL) ± IL-4 (10 ng/mL). Propidium iodide exclusion was used to determine the percent of viable cells at each stage of division. Summary of three independent experiments

The *Top2b*^*+/EE587E*^ heterozygous mice recapitulated the Hoffman syndrome patient defects in peripheral blood B cells without an effect on peripheral blood T cells (Fig. 6a, b; Table 1). To determine whether the remaining B cells in the heterozygous mice were functional, we compared *in vitro* activation of mutant B and T cells. In response to in vitro stimulation with LPS with or without IL-4, *Top2b*^*+/EE587E*^ derived B cells demonstrated decreased secretion of total IgG, IgG3, and IgG2b compared to littermate controls (Fig. 7a, Supplementary Fig. 7a, b). In contrast, in vitro stimulation of isolated splenic T cells showed similar IL-2 production in response to PMA and ionomycin in both *Top2b*^*+/EE587E*^ mice and controls (Fig. 7b). These results are consistent with the normal T-cell mitogen stimulation results observed in the patients (Table 1).

### B cells in Top2b heterozygotes have proliferation defects

B-cell proliferation and cell death has been modeled as a stochastic response during development and following antigenic stimulation^32,33^. Since *Top2b* is expressed at all stages of the cell cycle^34^, we asked whether Top2b affects the likelihood of stimulated B cells to proliferate rather than to undergo cell death. After three days in culture, stimulated mutant B cells were reduced in number (Fig. 7c), and had greater lactate dehydrogenase (LDH) release in response to LPS stimulation, compared to wild-type controls (Supplementary Fig. 7c). T-cell proliferation was unaffected (Fig. 7d). Splenic derived B cells were then cultured with LPS and IL-4 to enhance the rate of proliferation^35^ and assessed by CFSE dilution and propidium iodide to determine if mutant B cells were defective in cell division. After 5 days in culture, the majority of viable wild-type B cells had undergone 3–4 rounds of division, while more than 90% of mutant B cells did not advance beyond the first division (Fig. 7c, Supplementary Fig 7d.) Together these results further support a role for mutant Top2b in B cells, leading to an increased propensity to undergo apoptosis rather than cell division.

## Discussion

Genetically defined immunodeficiencies were first described with the identification of *BTK* mutations in X-linked (Bruton’s) agammaglobulinemia^36,37^, and subsequently expanded to include several B-cell receptor genes, including BLNK, µ heavy chain, and CD79A/B^5,38^. Given the complexities of B-cell development, numerous other genetic defects likely remain to be revealed. In three families with Hoffman syndrome^9,10^, we identified three heterozygous mutations affecting the TOPRIM, or DNA gating, domain of the type II topoisomerase TOP2B. Introduction of these mutations into *S. cerevisiae* indicate that the mutations are partially dominant loss-of-function mutations, consistent with the nonviability of the homozygous *Top2b*^*EE587E/EE587E*^ mice. Both the *Top2b*^*−/−*^ conditional knockout mouse model and the *Top2b*^*+/EE587E*^ heterozygous mouse model exhibit immunological defects affecting B cells, but not T cells, which correlate with the immunodeficiency observed in Hoffman syndrome patients and support that the *TOP2B* mutations underlie this syndrome. Thus, our findings define a class of B-cell immunodeficiency and are the first description of a defined monogenic syndrome due to mutations in the essential gene *TOP2B*.

Defects in *Top2b* in mice give rise to a unique B-cell defect. Unlike mutations in genes like *BTK* or *PIK3R1*, which give rise to a failure of cells to progress past a distinct stage in B-cell development^39^, loss of Top2b function in mice gives rise to reduced levels of B cells in multiple stages of B-cell development and defects in the production of specific immunoglobulins when challenged with antigens. Consistent with this, *Top2b* is highly expressed in B cells, and *Top2b*^*+/EE587E*^ and *Top2b*^*−/−*^ *mb1 cre* mice have much lower levels of expression of B cell-specific transcription factors, including *Pax5*. The relative loss of *Pax5* specifically would favor proximal gene usage, resulting in a reduced V gene repertoire, without affecting T-cell development^40^. *Top2b* is expressed at much lower levels in T cells, with numbers of T cells and T cell-specific transcription factor expression similar between wildtype, *Top2b*^*+/EE587E*^ and *Top2b*^*−/−*^ *mb1 cre* mice. In addition to reduced B cell numbers, B cells from *Top2b*^*+/EE587E*^ mice respond poorly to stimulation, producing less total IgG, IgG3, and IgG2 in response to LPS or LPS + IL-4. Mutant B cells also fail to proliferate or undergo cell death after stimulation; whereas T cells derived from *Top2b*^*+/EE587E*^ mice responded normally to PMA and ionomycin. Thus, defects in *Top2b* not only affect the number of mature B cells that survive checkpoints during development, but also the ability of the mature B cells to properly respond to stimulus.

Hoffman syndrome patients have no detectable B cells in the peripheral blood, nor form germinal centers, and patients did not generate protective serologic titers to immunization^9^; however, detectable amounts of circulating immunoglobulins indicate that some B cells are present, but are below the clinical limits of detection. In contrast, patients show normal numbers of CD4+ and CD8+ T lymphocytes and T-cell mitogen stimulation. These observations are consistent with the profound defects in B-cell numbers and stimulus response but normal T cells seen in the *Top2b* mutant mice. A bone marrow biopsy has not been clinically indicated for patients, leaving open the question of where in B-cell development the failure occurs in humans; however, the analysis of the *Top2b* mutant mice suggests that in the absence of functional *TOP2B*, the bone marrow generates a reduced B cell compartment and that the remaining surviving cells have reduced ability to respond to antigen or general stimuli. While our murine model has some phenotypic differences from our patients, it does provide opportunities to study B cells, which are not detected or practically assessed in our human patients.

*TOP2B* defects specifically cause a profound loss of the B-cell compartment and B-cell function. One possible explanation for this specificity is that long gene transcription is preferentially reduced when topoisomerases are absent or inhibited^2,3^, and that the murine B-cell transcription factors are notably longer (*EBF* is 390 kb, *Pax5* is 178 kb, and *Foxo1* is 85 kb) compared to T-cell transcription factors (*Tcf1* is 30 kb and *Notch1* is 45 kb) or shared transcription factors (*Rag1* is 11 kb and *E2A* is 24 kb). Consistent with this, *TOP2B* is more highly expressed in B cells than in T cells in both humans and mice (Fig. 6h, i)^28–31^.

Topoisomerases act to prevent the generation of free DNA breaks during transcription. In our murine models, the most obvious reduction in B cell numbers occurs during V(D)J recombination in the bone marrow, and during class switch recombination in the in vitro studies. The order of immunoglobulin gene rearrangement in B cells is predetermined, with heavy chain rearrangement preceding light chain rearrangement^41^. An inability to accurately form double strand breaks, failure to repair them or effectively pair recombination signal sequences would also be associated with inability to proliferate past the first replication. While these recombination defects can lead to immunodeficiency^42,43^, we might expect a greater tendency to malignancy, which has not been observed in our patients or mice.

TOP2B is also involved in transcriptional regulation during differentiation^44,45^ through association with multiple transcription factors, CTCF, and cohesin at transcriptional domain boundaries^46,47^ Analysis of published Top2b ChIP-seq data and etoposide-induced DNA breaks as determined by END-seq from murine primary B cells^48^ showed the presence of Top2b (but not Top2a) in the promoter and enhancer (intron 5) regions of *Pax5* (Supplementary Fig. 8). CTCF binding has also been described at multiple sites within the murine *Igh* locus, where it is required for distal gene usage with Pax5 and cohesion subunit Rad21, in a stage specific fashion^49^. Impairments in germline transcriptional activity, caused by defective Top2b activity could affect *Igh* locus accessibility, with negative effects on V(D)J recombination^50^. Similarly, in human cell lines, CTCF binding is prominent in the promotor and enhancer regions. The reduced numbers of NK cells in two of the patients (Table 1) may be consequent to similar binding patterns of Top2b to the *Nfil3* promoter, a regulator of NK cell differentiation^51–53^.

Given the potential for transcriptional defects to affect multiple organs, it is not surprising that other features characterize children with this syndrome since Top2b promotes correct expression of developmentally regulated genes^45,54,55^. We cannot, however, rule out the possibility that the TOPRIM domain mutations might also allow DNA breaks to accumulate. Consistent with this latter possibility, a brief clinical report recently described a patient with progressive microcephaly, developmental delay, and hypotonia, who had a variant outside of the TOPRIM domain of *TOP2B* (H58Y)^56^. However, no similarities to our patients were identified and it is currently unclear if this variant is causative. While beyond the scope of this manuscript, we expect these insights will ultimately help in unraveling the mechanism underlying the various aspects of Hoffman syndrome.

## Methods

### Human subjects

Blood samples were obtained from the affected patients and their immediate family members. All participants, or parents or legal guardians if the patient was a minor, provided written informed consent under protocols approved by the UCSD and/or CHOP Institutional Review Boards for participation in research and publication of data.

### DNA isolation, library construction, and sequencing

For families 1 and 2, genomic DNA was isolated from blood by the QIAGEN DNA extraction kit and randomly fragmented. Fragments of the desired length were gel purified. Adaptor ligation and DNA cluster preparation were performed with the library preparation kit per the manufacturer’s instruction (Illumina, San Diego). Whole genome sequencing was performed at Beijing Genomics Institute with the Illumina HiSeq2000 platform to a mean, per-sample depth of 30×.

### Sequence analysis

For families 1 and 2, all raw 100 bp paired-end reads were aligned to the human genome reference sequence (hg19) using BWA v0.5.9-r16^57^ with default parameters for paired-end reads except for seed length (-l) set to 35. Aligned reads were realigned using GATK’s^58^ IndelRealigner v 1.6-9-g47df7bb. Duplicate reads were removed using Picard Tools v 1.69 MarkDuplicates [http://picard.sourceforge.ne]. Finally, the GATK’s TableRecalibration tool was used to recalibrate the reads’ base quality scores. (Supplementary Tables 2 and 3).

SNPs and indels were called using GATK v 1.6-5-g557da77 UnifiedGenotyper with parameters -stand_call_conf 20.0, -stand_emit_conf 10.0, and -dcov 2000. UCSC assembly hg19 and dbSNP137^11^ were used as references. The called variants were recalibrated using the same GATK modules, VariantRecalibrator and ApplyRecalibration. SNPs and Indels were recalibrated separately as recommended by GATK’s best practices. For SNPs, we used the references HapMap v3.3, 1000G Omni2.5, and dbSNP137 and the annotation options QD, HaplotypeScore, MQRankSum, ReadPosRankSum, FS and DP during the VariantRecalibrator step. For Indels, we used the reference Mills 1000G gold standard indels from GATK and the annotation options DP, FS, ReadPosRankSum, MQRankSum, and HaplotypeScore during the VariantRecalibrator step. We then set the tranch filter level to 99 and 95 for SNPs and Indels, respectively. Since all samples were females, we removed variants called on the Y chromosome.

Variants were queried against dbSNP137 to determine novel or known variants. Next, we used snpEff^59^ v. 2.0.5 in combination with GATK VariantAnnotator, both with default parameters, to identify the different the functional and impact on coding genes (Supplementary Tables 3 and 4).

To identify variants that could be causal we applied the following filters: (1) the variant must have passed all variant calling filters, (2) the variant is not in dbSNP137, (3) the genotype was not called “undetermined” by GATK for any of the 5 samples (gt equal “./.”), (4) for Family 1, we removed any variant that was called reference in either of the samples or if it was called alternate in either of the unaffected controls in Family 2, and (5) for Family 2, we removed any variant that was called reference in the affected patient or nonreference in the unaffected controls. After applying the filters to select for a causal variant, there was only 1 gene with nonsynonymous changes in only the affected patients in both families, *TOP2B* (Fig. 1a).

For Family 3, after informed consent was obtained, the patient was recruited under research protocol approved by the institutional review board of the Children’s Hospital of Philadelphia. The sequencing was performed in a clinical laboratory and the resulting data were fed into our pipeline to exclude synonymous variants, variants with minor allele frequency greater than 0.5% in either the 1000 Genomes Project or the 6503 exomes from the National Heart, Lung, and Blood Institute Exome Sequencing Project (ESP6500SI), variants previously identified in controls by our in-house exome variant database (greater than 4000 individuals), and variants that were predicted by SIFT/PolyPhen-2 scores to be benign. After filtering, no potential candidate gene with paired mutations was identified. To facilitate identification of de novo dominant variants, we then presumed that the variants responsible for the disease would be extremely rare and probably absent in the general population due to the severity of the phenotype. Therefore, we selected for variants not present in 1000 Genomes Project, ESP6500SI, ExAC v0.3, and not identified in subjects by our in-house exome variant database. A variant in *TOP2B* was identified as the most likely candidate.

For all three families, mutations in *TOP2B* were confirmed by Sanger sequencing.

### *S. cerevisiae* strains

*S. cerevisiae* experiments used strains JN394t2-4^60^, RDKY2325 *(***MATa** *ura3-52, leu2Δ1, trp1Δ63, his3Δ200, lys2Δ202)*, RDKY2326 *(***MATα** *ura3-52, leu2Δ1, trp1Δ63, his3Δ200, lys2Δ202*), and RDKY3590 (**MATa** *ura3-52, leu2Δ1, trp1Δ63, hom3-10, lys2::InsE-A10*) were a gift from RD Kolodner (UCSD). Strains RDKY9075 (**MATα** *hom3-10, ura3 Δ0, leu2Δ0, trp1Δ63, his3Δ200, lyp1::TRP1, cyh2-Q38K, iYFR016C::PMFA1-LEU2, can1::PLEU2-NAT, yel072w:: CAN1-URA3, HUG1-EGFP::hphNT1, trp1::kanMX4)*, RDKY9073 (**MATa** *his3∆0 leu2∆0 met15∆0 ura3∆0 HUG1-EGFP::hphNT1)* and RDKY8176 (**MATα** *hom3-10*, *ura3Δ0, leu2Δ0*, *trp1Δ63*, *his3Δ200*, *lyp1::TRP1*, *cyh2-Q38K, iYFR016C::PMFA1-LEU2, can1::PLEU2-NAT, yel072w:: CAN1-URA3, HUG1-EGFP::hphNT1, rad27::HIS3*) were a gift from Binzhong Li and RD Kolodner (UCSD). The heterozygous *top2* diploid strain LBY0039 (**MATa/MATα** *ura3-52/ura3-52*, *leu2Δ1/leu2Δ1*, *trp1Δ63/trp1Δ63*, *his3Δ200/his3Δ200*, *lys2Δ202/lys2Δ202*, *top2::G418/TOP2*) was constructed by crossing RDKY2325 and RDKY2326 and knocking out one copy of *TOP2* by PCR-based gene disruption using standard techniques^61,62^. Derived strains are summarized in Supplementary Table 9.

### *S. cerevisiae* plasmids

Plasmids containing a chimeric *YTOP2*-*TOP2B* fusion in which the first five residues of yeast *TOP2* are fused to residues 46–1621 of human *TOP2B*^18,60^, or *YTOP2* were used. To avoid the toxicity of the *S. cerevisiae TOP2* in *E. coli* during plasmid propagation, an intron was inserted into the catalytic site of YTOP2, with the sequence GTATGT_gattttgaggctgtaacaattttctttcttttaaaaaaatgattaagttgaa_CACTAAC_atagtagaatcttca_CAG, modified from the *MCM21* intron (Supplementary Fig. 3)^63^. The length of 82 base pairs is not evenly divisible by 3, resulting in no possible readthrough without frameshift^64^. In addition, this modified *MCM21-PacI* intron has stop codons in all three reading frames. Briefly, genomic DNA was cloned from RDKY3590 using the following primers A: 5′-ACT CCA ACG TCA AAG GGC GAA AAA CCG TCT ATC AGG GCG ATG GCC CAC TAC CCG CGG AGG AAC CAG GGG CTA ACT GT-3′, B: 5′-GTC GAC gat tta at cat TTA ATT AAa aga AAG AAA ATT GTT ACA GCC TCA AAA TCA CAT ACC TTC TGT CAG AAC TAA AGT AC-3′; C: 5′-CCG CGG gat tct tTT AAT TAA atg att aag ttg aaC ACT AAC ata gta gaa tct tca CAG GGG ATT CCG CCT TGT CAT TAG-3′, D: 5′-GTG AAC CAT CAC CCT AAT CAA GTT TTT TGG GGT CGA GGT GCC GTA AAG CAC TGT CGA CTT CTC GGG CAG ATC TTT GT-3′. Fragments AB (5′) and CD (3′) were separately cloned into pCR2.1-TOPO via the manufacturer’s specifications. The 5′ and 3′ portions of TOP2 with intron were ligated, and subcloned into the *ARS-CEN* plasmid pRS314^65^. The presence of the intron in each of the plasmids was verified by sequencing by Eton Bioscience, Inc. (San Diego, CA). Mutations equivalent to the patient-identified mutations were introduced into the plasmids (Supplementary Table 8).

### *S. cerevisiae* complementation studies

Temperature sensitive strain JN394 *top2–4*^66^ was transformed with plasmids containing wild-type *TOP2*, *top2* mutant, or empty vector pRS314^62^. Yeast strains were grown in selective liquid culture at 25 °C, serially diluted and replica plated. Plates were incubated at 25 °C or 37 °C and viability assessed.

### *S. cerevisiae* sporulation

The diploid strain LBY0039 (**MATa/MATα** *ura3-52/ura3-52, leu2Δ1/leu2Δ1, trp1Δ63/trp1Δ63, his3Δ200/his3Δ200, lys2Δ202/lys2Δ202, top2::G418/TOP2*) was transformed with one of the vectors: ScTOP2, ScTOP2-EE587E, ScTOP2-S483L, ScTOP2-G633S, pRS314. Strains were sporulated in nitrogen-deficient starvation medium using standard techniques, and random spores isolated by lysing cells in 50 μl 1 mg/ml lyticase followed by incubating for 30 min at 37 °C. Spores were resuspended in sterile water, vortexed in Eppendorf tubes to adhere random spores to the walls of the plastic tubes, and washed with sterile water. Washed spores were resuspended in 1.5% NP40 and spread on nonselective YPD plates. Spore clones were then genotyped for growth on CSM–TRP medium (presence of the pRS314-derived vector), YPD+ 200 μg/mL G418 medium (presence of genomic *top2::G418*), and for mating type (Supplementary Table 6). When diploid strains containing the empty vector or *top2* mutant vectors were sporulated, a few **MATa** and **MATα** spores (*n* = 6–9) were observed that were tryptophan prototrophs and G418 resistant. Evaluation of these spore clones by PCR amplification of an internal region of the *TOP2* gene demonstrated that these spore clones likely retained a second copy of chromosome XIV due to chromosomal nondisjunction. These spore clones were not included in the analysis.

### Assessment of doubling time in *S. cerevisiae*

Diploid *S. cerevisiae strains* carrying wild-type or mutant *TOP2* were grown in selective liquid culture to absorbance of 1.0 at O.D. 600. Serial absorbance measurements were taken hourly, and doubling time calculated from the exponential growth phase. Growth studies were performed five independent times.

### Decatenation assay

*S. cerevisiae* strains carrying wild-type or mutant *TOP2* were grown to O.D. 600 2.5–3, and nuclei isolated per protocol http://www.biolchem.ucla.edu/labs/grunstein/Yeast%20Nuclei%20Isolation.html, modified from Ponticelli and Struh^67^. Briefly, strains were grown to an O.D._600_ = 2.5–3, and resuspended in buffer (1 M Sorbitol, 50 mM Tris pH 7.5, 10 mM MgCl_2_, 3 mM DTT) and Zymolase (Zymo Research) added to a final concentration of 60 U/ml. Cells were incubated at 30 °C with gentle shaking until 80% of cells formed ghosts. Extracts were washed and spheroplasts resuspended in YPD with 1 M sorbitol followed by washing with 1 M sorbitol. Extracts were mechanically homogenized in Buffer N containing 25 mM K_2_SO_4_, 30 mM HEPES (pH 7.6), 5 mM MgSO_4_, 1 mM EDTA, 10% glycerol and 0.1% NP-40 at 4 °C. The resulting homogenate was centrifuged at 5000×*g* for 15 min at 4 °C. The supernatant was reserved and centrifuged at 10,000×*g* for 25 min at 4 °C. The final pellet containing the isolated nuclei was resuspended in 40 µl Buffer N, aliquoted and stored at −80 °C. Protein concentrations were determined by Bradford assay (Bio-Rad) and 30 ng of extract used in the assays. Nuclei were incubated with 500 ng kinetoplast DNA (Topogen, Inc.) in the presence of ATP per instructions provided by Topogen, Inc. Reactions were incubated at 37 °C for 60 min and terminated with loading dye. Reactions were loaded in entirety on a 1% agarose + 0.5 μg/ml ethidium bromide gel for analysis of decatenation products, compared to the Topo II decatenated kDNA marker (Topogen, Inc. TG2020-1). Band area was quantified using Image J. Three independent assays were performed. A dilution assay using wild-type nuclear extracts (2.5–40 ng) was performed to confirm the linearity of the assay, and linear regression shown in Supplementary Fig. 4c.

### Mouse models

*Top2b*^*flox2*^ mice were provided by Y.L. Lyu (via C-M Chuong, USC Keck School of Medicine, Los Angeles, USA)^68^. As *Top2b*^*−/−*^ mice are not viable^21^, *Top2b*^*flox2*^ mice were bred to various mouse lines expressing conditional Cre recombinase, to evaluate B-cell populations. Mice were bred to *Cd79a*^*tm1(cre)Reth*^ (herein termed *mb1-cre)* kindly provided by M. Reth (University of Freiburg, Germany)^20^ via R. Rickert (Prebys Medical Discovery Institute, La Jolla, USA) or *Cd19*^*tm1(cre)Cgn*^ mice (*cd19-cre*, The Jackson Laboratory, USA). Additionally, *Top2b*^*+/EE587E*^ mice were generated to express the delAGA mutation on mouse *Top2b* (see below). All mice were between 12 and 22 weeks of age with age-matching within each experiment; both male and female mice were used. No additional randomization was performed, no animals excluded from analyses, and no additional blinding performed. Experimental protocols were approved by the Institutional Animal Care and Use Committee at the University of California, San Diego.

### Generation of *Top2b*^*−/−*^ mb1-cre and *Top2b*^*−/−*^ cd19-cre mice

Conditional *Top2b*^*−/−*^*mb1* and *Top2b*^*−/−*^*cd19* mice were generated by breeding *Top2b*^*flox2/flox2*^ animals with *mb1-cre or cd19-cre* mice producing offspring that are *Top2b*^*+/Δ2*^*mb1-cre* and *Top2b*^*+/Δ2*^*cd19-cre*, respectively. These mice were then crossed with *Top2b*^*flox2/flox2*^ mice giving progeny of the desired genotypes; *Top2b*^*Δ2/Δ2*^*mb1-cre (Top2b*^*−/−*^*mb1)* and *Top2b*^*Δ2/Δ2*^*cd19-cre* (*Top2b*^*−/−*^*cd19*). qPCR for *Top2b* expression in isolated B cells confirmed an absence of *Top2b* expression (Source data are provided in Source Data file).

### Generation of Top2b+/delAGA knockin mice

A conditional by inversion (COIN) allele to express the delAGA mutation on mouse *Top2b*, with the potential to revert to wild-type sequence via inducible cre-lox technology, was created as follows: a segment of human *TOP2B* containing the intron 13 splice acceptor (SA) region, exon 14, and intron 14 splice donor (SD) region was synthesized in reverse orientation (reverse [*TOP2B* int13 SA-ex14-int14 SD]). This fragment was ligated to a synthetic region consisting of the mouse *Top2b* intron 13 SA, exon 14 containing the del AGA allele, and intron 14 SD, in forward orientation (forward [*Top2b* int13 SA-ex14 del AGA-int14 SD]). To reduce the potential for hairpin formation between human and mouse exon 14 base pair sequences, multiple silent mutations were included in the reversed human exon 14. LoxP and Lox2372 recombination sites were interspersed between human and mouse sequence, to mediate later COIN flipping and cre recombinase-mediated deletion^69^. A reverse orientation neomycin phosphotransferase (npt) artificial minigene, flanked by flippase recognition target sites^70^, was ligated 5′ to the human segment. Expression of *npt* in mammalian cells was driven by the promoter region of the human ubiquitin C (*UBC*) gene^71^ (coordinates 125398319:125399530 in the antisense strand of human chromosome 12; Human CCDS set: CCDS9260), whereas expression in *E. coli* was driven by the EM7 promoter (Invitrogen). The npt open reading frame was followed by the 3′ region of the mouse *Pgkl* gene, containing a polyA signal and associated sequences (coordinates 103398979–103399440 of chromosome X), to ensure proper message termination. In addition, Rox recombination sites^72^ were introduced to flank the npt minigene and reverse [*TOP2B* int13 SA-ex14-int14 SD]. The final sequence of elements in the COIN module is: loxP-Rox-Frt-reverse [*UBC*-neo-*Pgkl* polyA]-Frt-lox2372-reverse [*TOP2B* int13 SA-ex14-int14 SD]-reverse (loxP)-Rox- forward [*Top2b* int13 SA-ex14 del AGA-int14 SD]-reverse (lox2372).

The synthetic fragment described above was used to modify a mouse bacterial artificial chromosome (BAC) using Velocigene technology^73^ and bacterial homologous recombination^74^. The recombined BAC was sequence-verified, linearized, and electroporated into mouse embryonic stem cells (F1H4:50% C57BL/6NTac/50% 129S6/SvEvTac, Regeneron Pharmaceuticals). Appropriately targeted stem cell clones were identified by loss of allele (LOA) assay^73^. The npt minigene was then excised by Flpe^75^.

Qualitative rtPCR analysis on Flpe-treated stem cell clones showed a population of COIN transcripts in which *Top2b* exon 14 (containing delAGA) was skipped. To obtain a clean allele, the COIN module was excised using the Dre recombination system, leaving exon 14 delAGA intact. The final allele is as follows: loxP-Rox-forward [*Top2b* int13 SA-ex14 del AGA-int14 SD]-reverse (lox2372). Correctly recombined ES cell clones were microinjected into 8-cell embryos from Charles River Laboratories Swiss Webster albino mice, yielding F0 VelociMice® that were 100% derived from the targeted cells^76^. Resulting F0 mice were subsequently bred to C57BL/6NTac. Top2b+/delAGA knockin mice are owned by Regeneron Pharmaceuticals, Inc., and any request for these mice should be directed to Regeneron Pharmaceuticals, Inc.

### Reverse transcription and quantitative PCR

RNA was isolated from transformed *S. cerevisiae*, murine spleen, or murine bone marrow using Trizol (Life Technologies) and cDNA was synthesized using Taqman Reverse Transcription reagents (Applied Biosystems), both per manufacturer’s instructions. For kappa-deleting recombination excision circles (KRECs)^22^, T-cell receptor excision circles, and yeast strain plasmid number analyses, DNA was isolated using Trizol (Life Technologies) or Qiagen’s yeast protocol, respectively. Quantitative PCR was performed using 100 nM each forward and reverse primer and iQ SYBR Green supermix (Bio-Rad) with a Bio-Rad CFX96 Real-Time System. Reaction parameters were as per Bio-Rad instructions with 3 min polymerase activation and DNA desaturation (95 °C), denaturation for 15 s (95 °C) and annealing/extension for 30 s at 55–60 °C, for 40 cycles. For KREC reactions, the cycle threshold was set at 0.03, and the *C*_T_ values of the coding joint and the signal joint compared for each sample^22^. For TREC reactions, a dilution series of mTREC plasmid molecules was used to establish a standard curve (100–10,000,000 molecules range), with annealing/extension time increased to 1 min^77^. The mouse sjTREC standard was a generous gift from Dr. Gregory D. Sempowski (Duke Human Vaccine Institute, Durham, NC, USA). Sequences for qPCR primers are shown in Supplementary Table 10. Data were visualized with CFX Manager v3.0 software.

### Flow cytometry

Peripheral whole-mouse blood was obtained by cheek venipuncture, and collected in EDTA-treated collection tubes. Red cells were lysed using ACK Lysing Buffer (ThermoFisher Scientific) per the manufacturer’s instructions. Totally, 10^6^ cells were stained with conjugated monoclonal antibodies to CD3 (17A2, 17-0032-80, 1:200), CD19 (1D3, 12-0193-81, 1:400), and B220 (RA3-682, 11-0452-82, 1:200; all from eBioscience, Inc.; used per manufacturer’s instructions). Murine spleens were mechanically dissociated and erythrocytes removed by hypotonic lysis. 1 × 10^6^ cells were stained with conjugated monoclonal antibodies to CD19 (1D3, 12-0193-81, 1:400), B220 (RA3-682, BioLegend, 130-102-357, 1:50), IgM (121-15F9, 48-5890-82, 1:100), IgD (11-26, 17-5993-80, 1:200), Flt3 (A2F10, 46-1351-82, 1:100), GL-7 (GL-7, 53-5902-80, 1:200), CD23 (B3B4, 12-0232-81, 1:200), CD38 (90, 12-0381-81, 1:200), CD21/35 (8D9, 47-0211-80, 1:100), MHC class II (M5/114.15.2, 12-5321-81, 1:100), CXCR4 (2B11, 147-9991-80, 1:200), (all from eBioscience, Inc., unless otherwise noted) per manufacturer’s instructions. Bone marrow cells were harvested from femurs and tibiae by flushing with PBS, and similarly stained with CD19 (1D3, 17-0193-80, 1:400), B220 (RA3-682, BioLegend, 130-102-357, 1:50), IgM (121-15F9, 48-5890-82, 1:100), CXCR4 (2B11, 147-9991-80, 1:200), Flt3 (A2F10, 46-1351-82, 1:100), CD43 (R2/60, 11-0431-81, 1:200), BP-1 (6C3, 12-5891-81, 1:200), CD25 (PC61.5, 61-0251-80, 1:200), CD24 (M1/69, 25-0242-80, 1:200) (all from eBioscience, Inc., unless otherwise noted). CFSE [5-(and 6)-carboxyfluorescein diacetate succinimidyl ester] staining was performed per manufacturer’s protocol (eBioscience, Inc.). Samples were acquired with a BD Biosciences LSR II cytometer using FACSDiva software, and analyzed with FlowJo V10 software. Gating strategy is shown in Supplementary Fig. 9^78^.

### Immunocytochemistry

For histological analysis, spleens were removed and post-fixed in 4% paraformaldehyde in 0.1 M PBS (pH 7.4) overnight at 4 °C, then cryoprotected for 24 h at 4 °C in 0.1 M phosphate buffer containing 20% sucrose. Sections measuring 30 μm thick were cut at −18 °C on a cryostat microtome (Cryocut, 1800), and collected free-floating in 0.1 M PBS containing 0.02% sodium azide. The spleen sections were first incubated for 60 min at room temperature in 0.1 M PBS containing 5% normal donkey serum and 0.8% Triton X-100. Sections were then incubated overnight at 4 °C with primary antibodies diluted in same solution used for pre-incubation. In order to perform triple immunolabeling, the following directly conjugated primary antibodies were used: (1) anti-CD3 (10 µg/mL, Alexa Fluor 488, clone 17A2, Biolegend, 100210), (2) anti-B220 (1 µg/mL, eFluor® 570 CD45R, clone RA3-6B2, eBioscience, Inc., 41-0452-80), and (3) anti-CD169 (5 µg/mL, Alexa Fluor 647, clone 3D6.112, BioLegend, Inc., 142408). The following isotype controls: (1) Alexa Fluor 488 Rat IgG2b, kappa (BioLegend, Inc., 400625), (2) eFluor 570 Rat IgG2a, kappa (ThermoFisher Scientific, Inc., 41-4321-82), and (3) Alexa Fluor 647 Rat IgG2a, kappa (BioLegend, Inc., 400526) antibodies were used for the negative control staining. 4′-6-diamidino-2-phenylindole (DAPI, 4 μg/mL, D3571, Invitrogen) was used for general nuclear staining of all sections. After washing three times for 10 min with PBS, sections were mounted on slides, air-dried, and then cover-slipped with ProLong Gold (ThermoFisher Scientific, Inc.).

### Image processing and quantitative assessment

The quantitative analysis utilized 4 age-matched control and 4 *Top2b*^*−/−*^ *mb1 cre* mice. The sizes of the splenic white pulp and its three sub-compartments were evaluated in at least ten follicles across three sections (500 μM apart) per mouse. The triple labeled sections visualizing CD3, B220, and CD169 positive cells were scanned via confocal microscopy (LSM 710 NLO Zeiss; ZEN 2010). The marginal zone (MZ), which contains B220-positive B cells and CD169-positive metallophilic macrophages, the transitional B-cell rich follicular region (FR) and CD3-positive T-cell rich periarteriolar lymphoid sheath subcompartments were measured by Image Pro-Plus (Media Cybernetics) software. All histological and image analyses were performed by blinded investigator.

### Immunizations

For ovalbumin immunizations, adult mice aged 12–22 weeks were immunized intraperitoneally with 50 μg of OVA (Worthington Biochemical) adsorbed to 1 mg of alum (Sigma-Aldrich) in 200 μl normal saline, administered as 2 injections given 7 days apart^79^. Seven days after the second injection, mice were anesthetized (ketamine 60 mg/kg plus xylazine 10 mg/kg intraperitoneally), the peritoneal and thoracic cavities opened, and blood samples obtained via cardiac puncture. Immunizations were performed in 3–4 independent experiments to permit age-matching within each experiment, with mice from two litters per experiment. Immunizations with Pneumovax®23 were performed by immunizing adult mice intraperitoneally once with 10 µl of Pneumovax®23 in 100 µl sterile PBS^27^. Mice were bled 24 h prior, and 6 and 42 days after immunization. Serum was isolated, and ELISAs performed as described below.

### Enzyme-linked immunosorbent assay (ELISA)

Total mouse IgG was assessed using the Ready-Set-Go® kit (ThermoFisher Scientific, Inc.) per manufacturer’s protocol. Quantification of OVA-IgG (Cayman Chemical, Ann Arbor, MI, USA) was performed by ELISA according to manufacturer’s instruction. Anti-Pneumovax®23 IgM and IgG subclass ELISAs were performed by coating Nunc ELISA plates with 5 μl Pneumovax® 23 in 50 μl PBS overnight. Plates were blocked for 2 h with 1% BSA, 0.1% sodium azide in PBS before adding 50 μl of diluted serum (1:80) to each well. After a 2 h incubation, anti-mouse IgM-HRP (1:1000, ThermoScientific, 62–6820), goat anti-mouse IgG3 (1:1000, ThermoScientific, M32707) or goat anti-mouse IgG2b (1:1000, ThermoScientific, M32407) were added for 2 h, followed by TMB substrate. Absorbance was measure at 450 nm^27^. Measurement of mouse IL-2 was performed by ELISA (R and D Systems) according to manufacturer’s instruction.

### Cell isolations

Magnetic bead isolation of cells from murine bone marrow and spleens was performed with the following Miltenyi Biotec kits: anti-B220 microbeads, Pan T-cell isolation kit, CD138+ microbeads, direct lineage depletion kit, CD117 microbeads, and anti-rat IgG microbeads. In all cases, isolations were performed per manufacturer’s protocol.

### Lymphocyte stimulation assays

B cells were isolated from murine spleens using anti-B220 magnetic beads per manufacturer’s protocol (Miltenyi Biotec) with >98% purity achieved. B cells were washed and then cultured in triplicate at 5 × 10^4^ cells/mL in RPMI 1640 + 10% fetal bovine serum, with 25 μg/mL LPS (*E. coli* 0111:B4, Calbiochem) with or without recombinant mouse IL-4 (10 ng/mL, Life Technologies)^80^. Cell counts were performed at day 3 of stimulation using BioRad TC20 automated cell counter. Supernatants were collected for measurement of immunoglobulins (see ELISA section above), or LDH release per manufacturer’s protocol (Pierce LDH Cytotoxicity Assay Kit, ThermoScientific). A subset was stained with CFSE [5-(and 6)-carboxyfluorescein diacetate succinimidyl ester] per manufacturer’s protocol (eBioscience, Inc.).

T cells were isolated from murine spleens using Pan T-cell isolation magnetic beads per manufacturer’s protocol (Miltenyi Biotec) with >98% purity achieved. T cells were washed and then cultured at 10^6^ cells/mL in RPMI 1640 + 10% fetal bovine serum and stimulated with 20 ng/mL phorbol 12-myristate 13-acetate (PMA) and 1 μg/mL ionomycin. Supernatants were collected for measurement of IL-2 (see ELISA section above).

### Comet assay

A neutral comet assay was performed as described by Olive and Banath^81^ with the following modifications. Frosted end microscope slides were washed, rinsed in methanol, and coated with 1% normal melting temperature agarose. After cooling, 4 × 10^5^ splenic B or T cells (isolated as above) were resuspended in 1% low-gelling temperature agarose, and applied to precoated slides. Cells were lysed in buffer containing 2% sarkosyl, 0.5 M Na_2_EDTA and 0.05% Triton-X at 4 °C for 1 h, followed by electrophoresis in Tris/Borate/EDTA buffer for 25 min at 0.6 V/cm. Slides were stained with 10 μg/mL propidium iodide. Fluorescent images were immediately acquired using a Leica TCS SPE laser-scanning confocal microscope at ×40 magnification (Leica Microsystems, Buffalo Grove, IL), with at least 5 fields and 50 cells captured per sample. Comet head and tail length were measured with ImageJ software.

### Immunoblot

B and T cells were isolated from wild-type spleens using magnetic beads as above. Cell lysates were prepared from 3 × 10^6^ cells by incubating with lysis buffer (50 mM Tris HCl pH 7.8, 150 mM NaCl, 0.1% NP-40, 1 mM PMSF) and denatured in Laemmli buffer (Bio-Rad, Inc.) with 355 mM 2-mercaptoethanol. After boiling, proteins were separated by SDS-polyacrylamide gel electrophoresis, transferred to polyvinylidene fluoride membranes, and immunoblotted with mouse monoclonal antibody to Top2b (1 μg/mL, ThermoFisher Scientific, MA5-24310) followed by horseradish-peroxidase conjugated anti-mouse (7076S, Cell Signaling Technology, Inc.), for detection by chemiluminescence (ThermoFisher Scientific). Anti-GAPDH-HRP was used to assess gel loading (1:3000, Proteintech, HRP-60004).

### Statistical analyses

Statistical analyses and graphing were performed in Microsoft Excel and Graphpad Prism (version 5.03; Graph Pad, Graph Pad Software Inc., CA) programs with the two-tailed, unpaired Student's *t* test, or the Kruskal–Wallis test, in all cases comparing wild-type vs. mutant. Flow cytometry data were analyzed by FlowJo software. Data are expressed as mean ± SEM. No samples or animals were excluded from the analyses, and no randomizations performed. A *p* value less than 0.05 was considered statistically significant.

### URLs

For Burrows–Wheeler aligner and Picard, see http://broadinstitute.github.io/picard/; for Genome Analysis Toolkit (GATK), see https://software.broadinstitute.org/gatk/.

### Reporting summary

Further information on research design is available in the Nature Research Reporting Summary linked to this article.

## Supplementary information

Supplementary Information

Reporting Summary

Source Data

## Data Availability

The authors declare that the data supporting the findings of this study are available within the paper and its Supplementary Information. Data contained in ChIP-seq and End-seq BED files (available from GEO, accession GSE99197)^48^ were visualized using the Integrated Genomics Viewer (IGV 2.4.8) Broad Institute. Genome and exome data for individual patients cannot be made publicly available for reasons of patient confidentiality. Qualified researchers may apply for access to these data, pending University of California, and CHOP institutional review board approval. Source data for Figs. 2d and 6i are included in the Source Data file.
